# Supplementary material for: A natural tandem array alleviates epigenetic repression of IPA1 and leads to superior yielding rice
Source: Nat Commun. 2017 Mar 20;8:14789. doi: 10.1038/ncomms14789 (PMC5364388; doi:10.1038/ncomms14789)
Supplement: Supplementary Information — Supplementary Figures and Supplementary Tables [file ncomms14789-s1.pdf]

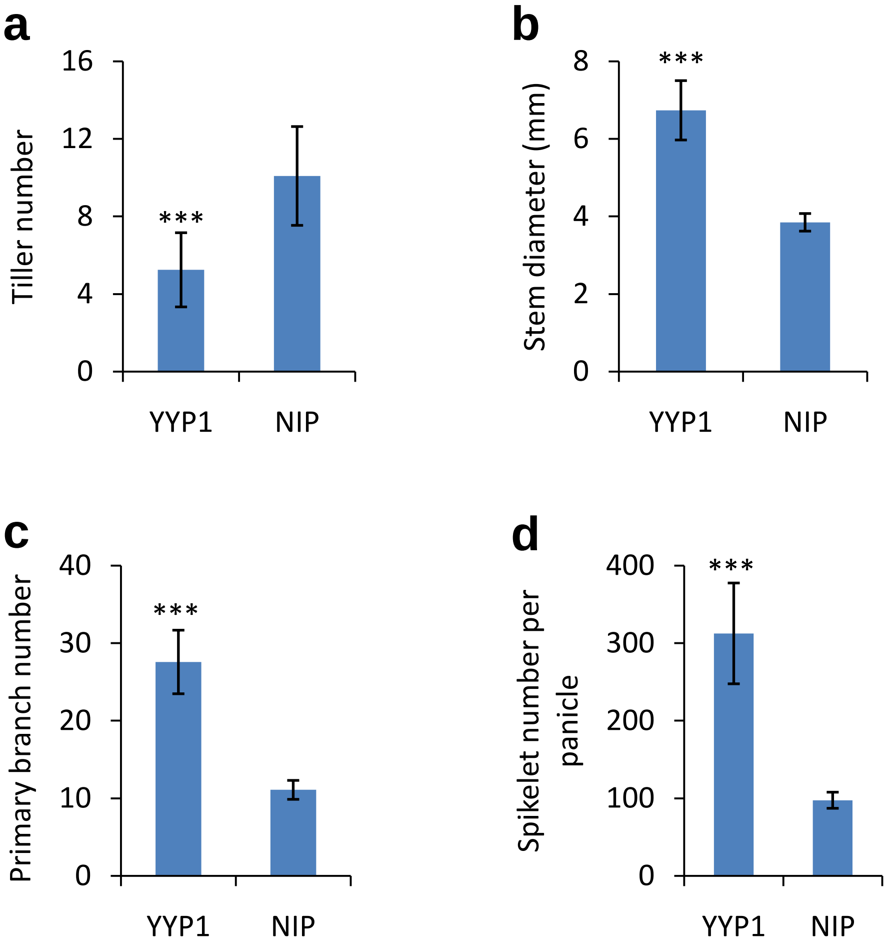

Supplementary Figure 1 YYP1 bears typical IPA characteristics in comparison with the conventional variety NIP. Columns shown here are (**a**) tiller number, (**b**) stem diameter, (**c**) panicle primary branch number and (**d**) spikelet number per main panicle. Values are means ± s.d. (n = 12). The triple asterisks represent significant difference determined by the Student's t-test at $P < 0.001$.

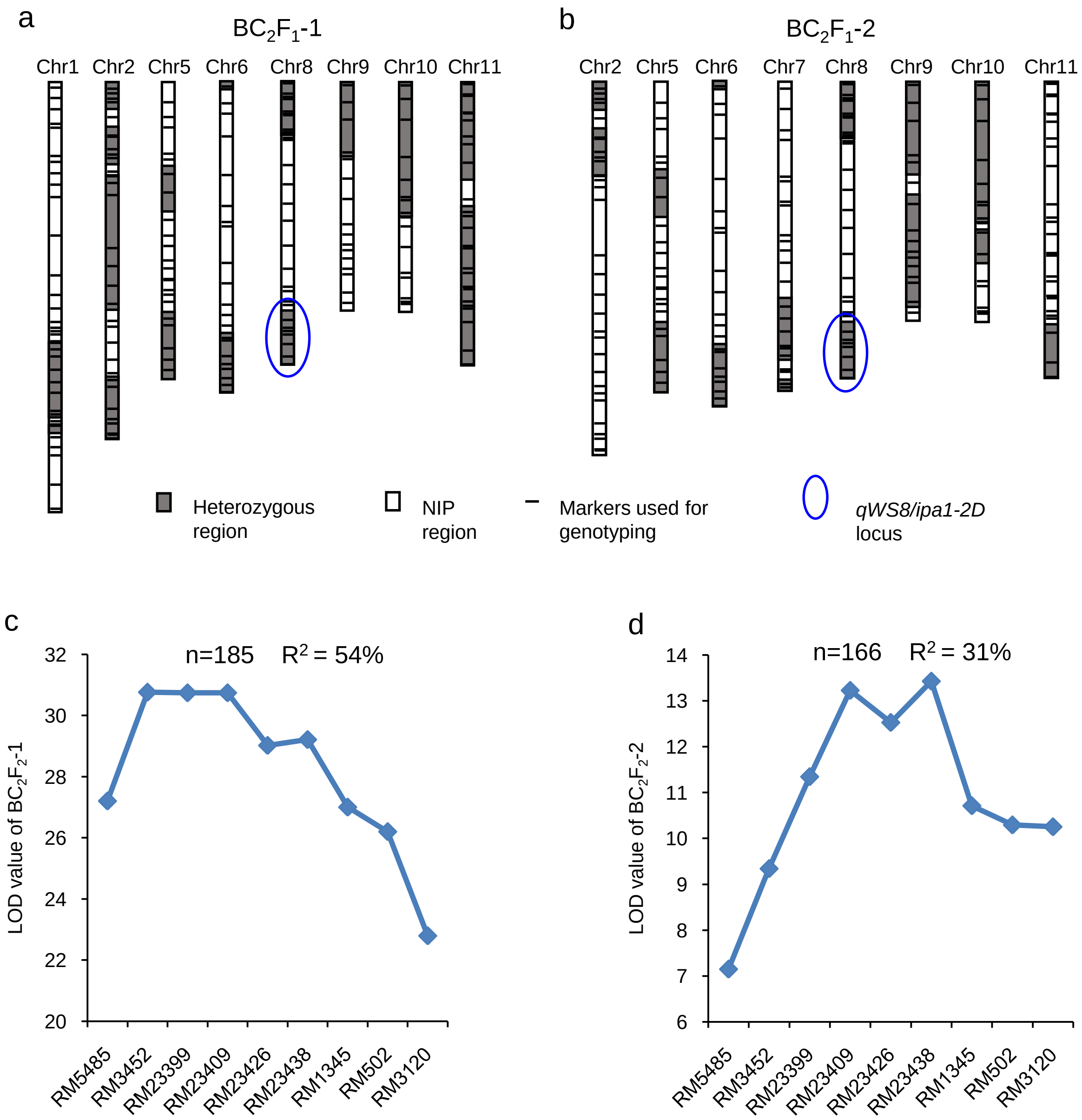

Supplementary Figure 2 Linkage analysis of two BC_2F_2 populations. (**a**) Genetic background of BC_2F_1-1 plants. (**b**) Genetic background of BC_2F_1-2 plants. Two plants shown in **a** and **b** were genotyped by genome-wide molecular markers and used to generate two BC_2F_2 populations for QTL confirmation and coarse mapping. Introgression of the YYP1 region is indicated by gray boxes, and NIP region is indicated by white boxes. Blue circles denote the position of *qWS8/ipa1-2D*. Only chromosomes with YYP1 introgression in the plants are shown. (**c**) Linkage analysis of the BC_2F_2-1 population. (**d**) Linkage analysis of the BC_2F_2-2 population. Marker-trait association was performed with nine markers to calculate the LOD value. The number of each population is showed and R^2 calculated by one-way ANOVA denotes the largest trait variation explained by the markers.

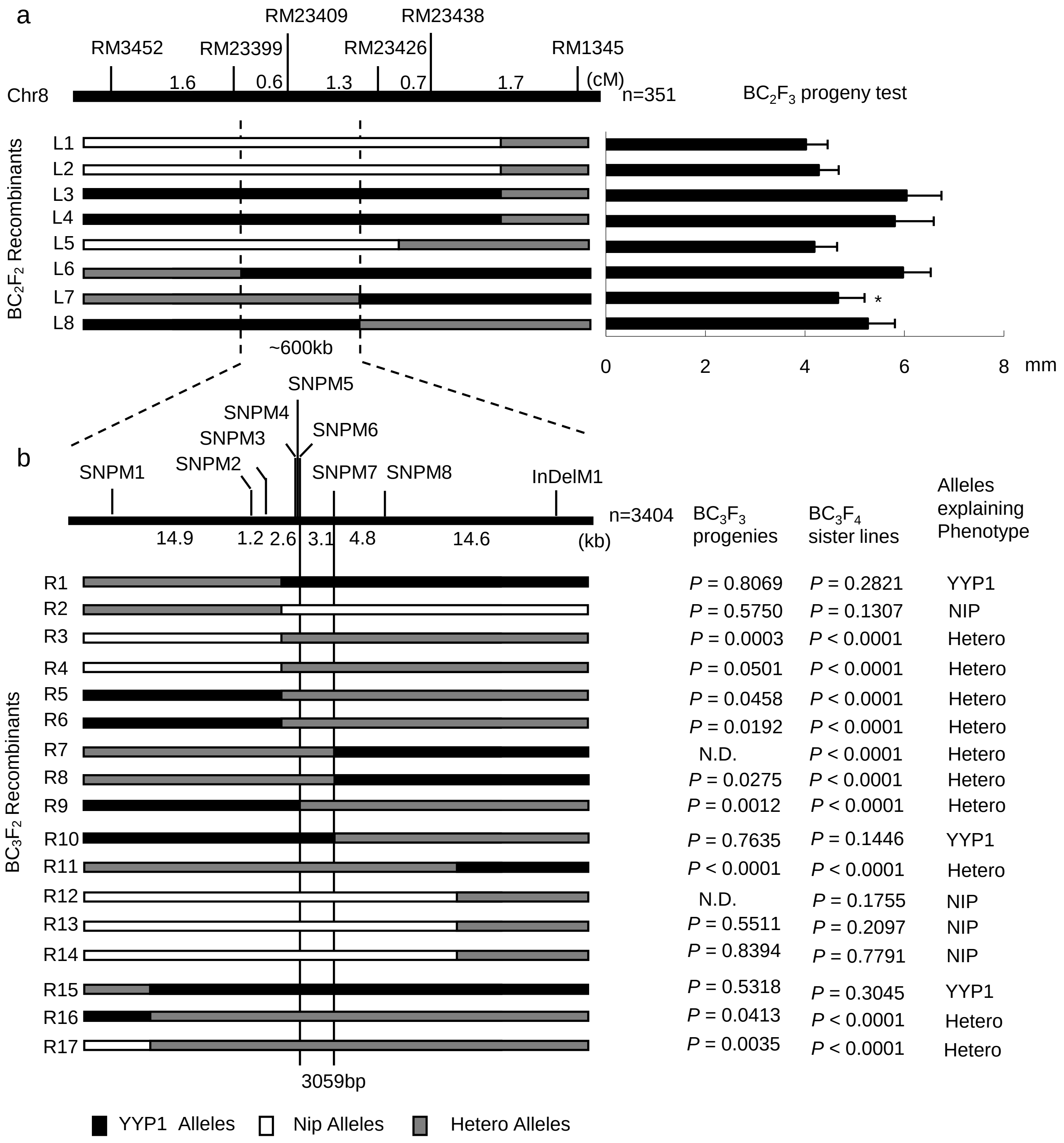

Supplementary Figure 3 Coarse mapping and fine mapping of *qWS8/ipa1-2D*. (**a**) Genotypes of BC_2F_2 recombinants and trait confirmation by BC_2F_3 evaluation that shows the stem diameter of internode 3 (right panel). Dashed lines denote the coarse mapping region. Asterisk indicates trait segregation coinciding with genotype segregation. (**b**) Genotypes of BC_3F_2 recombinants and trait confirmation by BC_3F_3 and BC_3F_4 evaluation. Solid lines denote the fine mapping region in NIP reference genome. The genotype segregation of individuals in BC_3F_3 line is determined by markers in the heterozygous region. One-way ANOVA was used to generate the *P*-value, and significant *P*-value ($P < 0.05$) indicates that the heterozygous region confers IPA trait segregation. N.D., not determined. The result was confirmed by BC_3F_4 homozygous sister lines with more significant *P*-value ($P < 0.0001$) determined by Student's *t*-test.

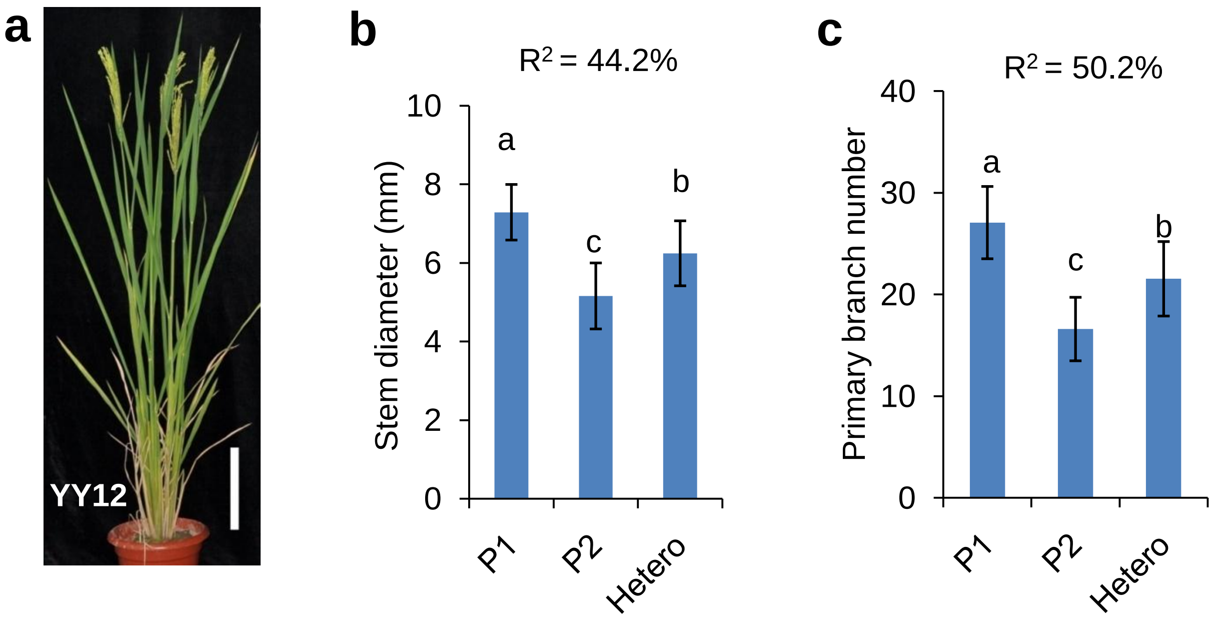

Supplementary Figure 4 Contribution of *qWS8/ipa1-2D* to the IPA determination of YY12. (**a**) Morphology of super hybrid rice YY12 widely cultivated in China. As the hybrid contains the heterozygous *qWS8/ipa1-2D* allele, the F_2 seeds were planted to perform single marker analysis. Scale bar, 20 cm. (**b,c**) Effect of the *qWS8/ipa1-2D* locus in determining IPA traits of stem diameter (**b**) and panicle primary branch number (**c**). Values are means ± s.d. (n = 15, 25 and 49 for P1 [YYP1 type], P2 [NIP type] and heterozygous alleles respectively). Different letters at top of each column indicate a significant difference at $P < 0.05$ determined by Tukey HSD test. Phenotypic variation explained by *qWS8/ipa1-2D* is shown as R^2 calculated by one-way ANOVA.

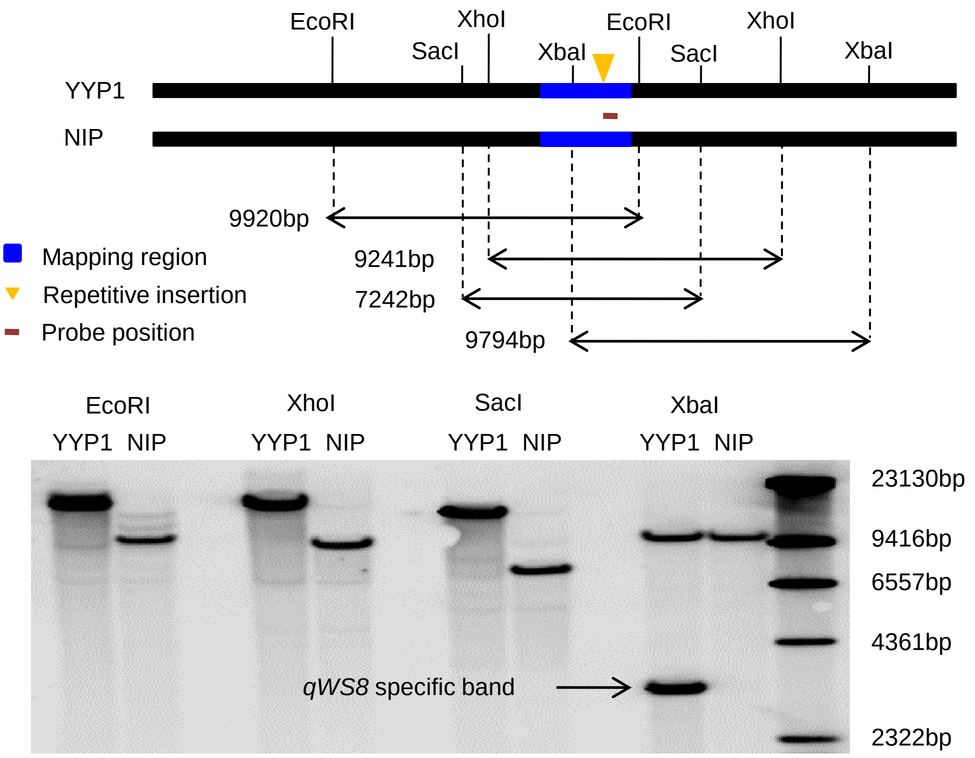

Supplementary Figure 5 Identification of an insertion underlying *qWS8/ipa1-2D* by southern blotting. According to the NIP genome, four sets of enzymes covering the mapping region were selected to performed the DNA digestion. Positions of the enzyme sites and probe are indicated on the sketch map. Blue indicates the 3-kb region uncovered by mapping and orange triangle indicates the cryptic insertion. The fragment size digested by each set of enzyme is labeled by double arrowhead corresponding to the NIP genome. Note that an additional band was detected by XbaI digestion in YYP1 (highlighted by arrow on Southern blot). DNA markers are shown at right.

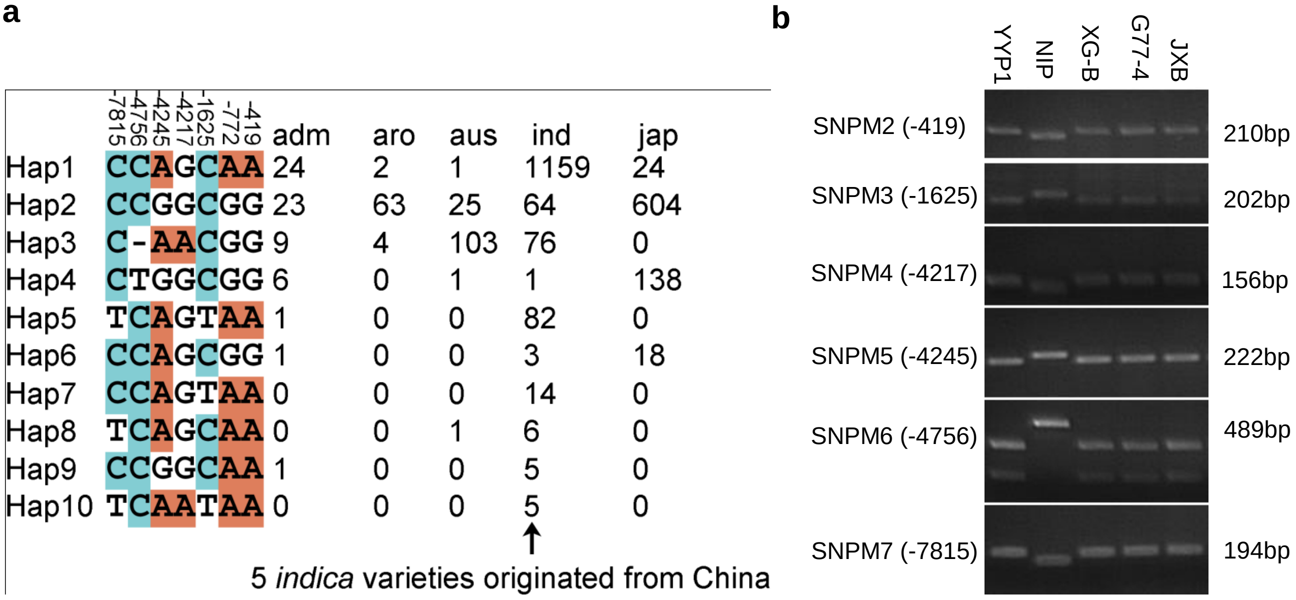

Supplementary Figure 6 Identification of *qWS8/ipa1-2D* haplotype from 2,464 rice varieties. (**a**) The ten haplotypes identified from the collections of varieties. Position of each SNP is denoted as distance from *IPA1* ATG (0). All the varieties were categorized into five subgroups. adm, admixed type; aro, basmati/sadri type; aus, aus/boro type; ind, *indica* type; jap, *japonica* type. Note that most of *indica* varieties belong to haplotype 1 and most of *japonica* varieties belong to haplotype 2. Only five *indica* varieties originated in China share exactly the same SNP with YYP1, and among them Xiangai-B, GENG77-4 and Jinxibai were selected for further analysis. (**b**) Confirmation of SNP haplotypes of Xiangai-B, GENG77-4 and Jinxibai. Six dCaps or Caps markers designed according to the SNPs were used to genotype the three varieties together with YYP1 and NIP. Numbers in parentheses indicate SNP positions, and sizes of the amplified fragments were shown on the right.

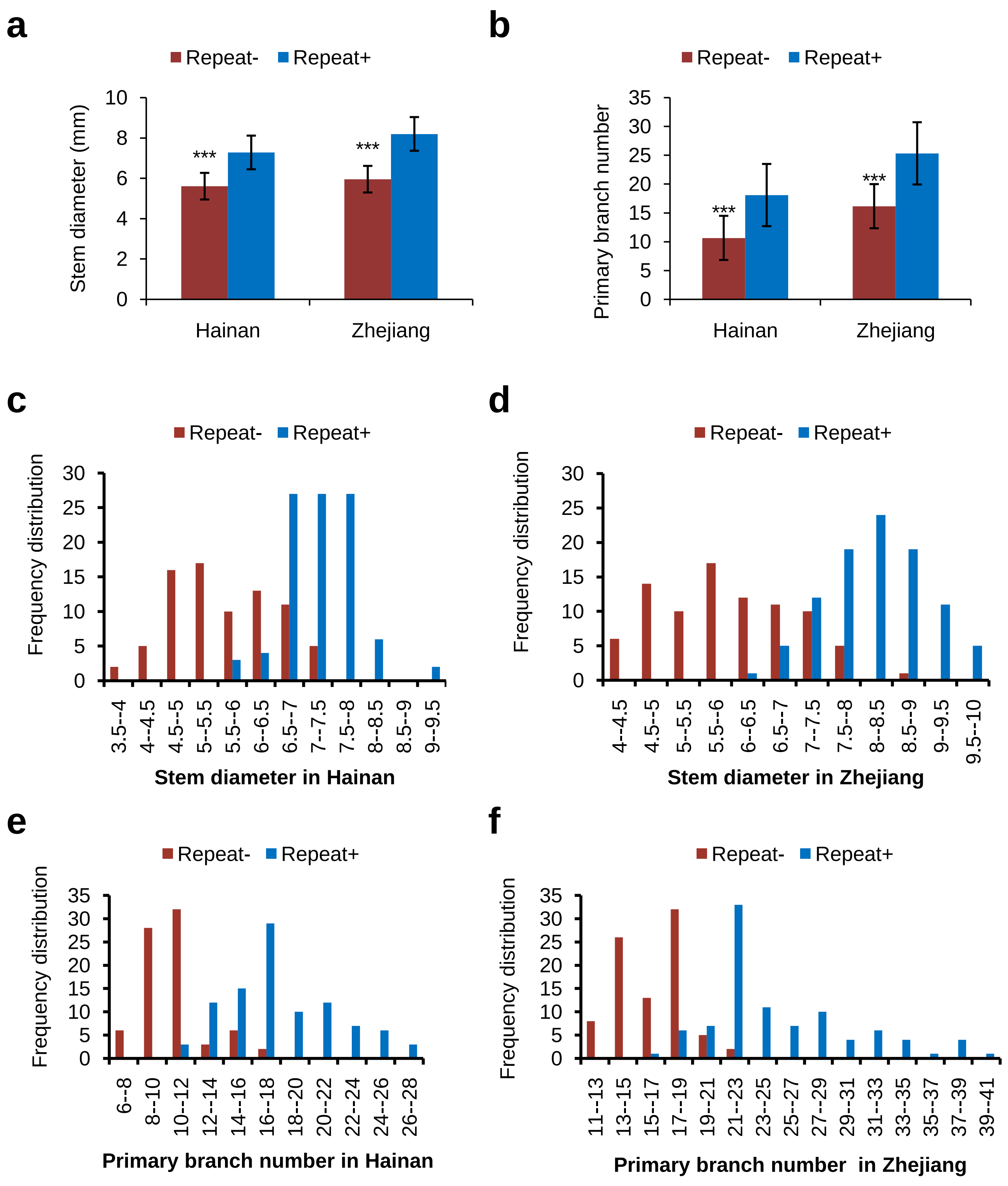

Supplementary Figure 7 Trait variations of modern IPA varieties with or without *qWS8/ipa1-2D* repeats in Hainan and Zhejiang. Columns shown here are (**a**) average stem diameter and (**b**) average panicle primary branch number of varieties. Values in (**a**) and (**b**) are means ± s.d. Triple asterisks represent significant difference between varieties with (+) and without (-) *qWS8/ipa1-2D* repeats determined by the Student's *t-test* at $P < 0.001$. (**c**,**d**) Frequency distribution of varieties for stem diameter in the Hainan station (**c**) and Zhejiang station (**d**). (**e**,**f**) Frequency distribution of varieties for panicle primary branch number in the Hainan station (**e**) and Zhejiang station (**f**).

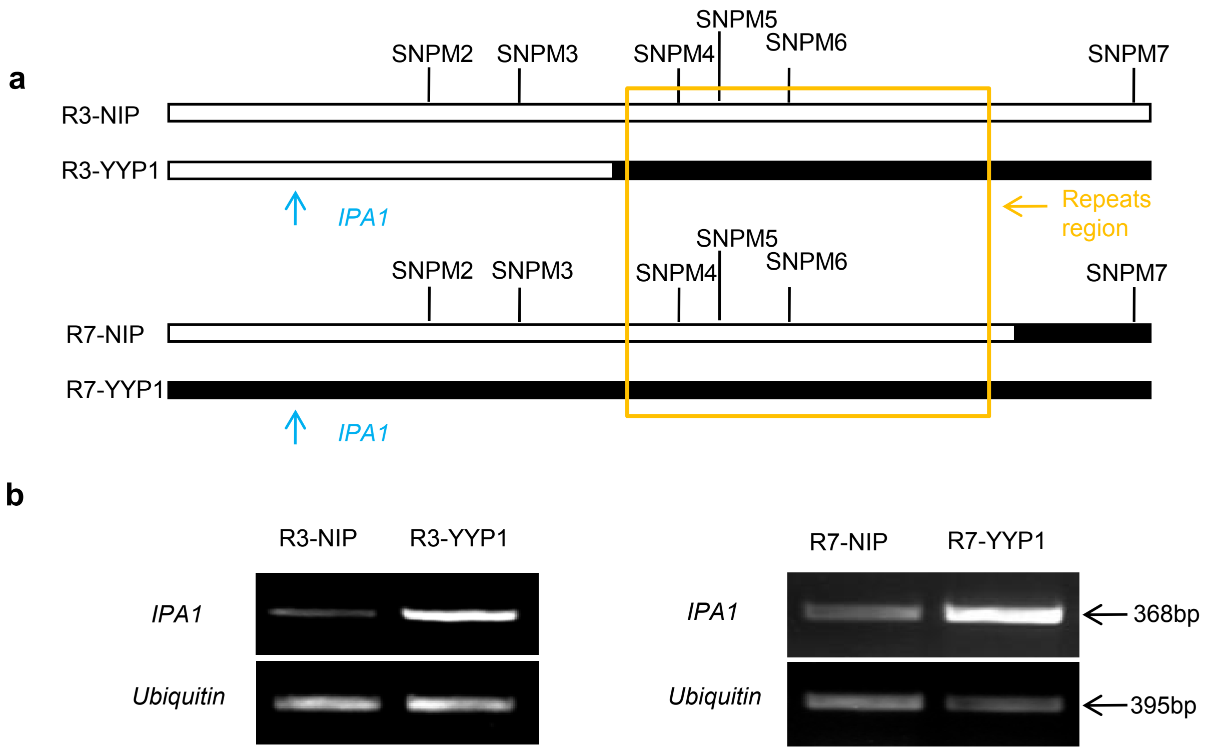

Supplementary Figure 8 Expression levels of *IPA1* in recombinant sibling lines. (**a**) Schematic map denoting genotypes of sibling lines from two recombinants. Black and white boxes indicate genomic region from YYP1 and NIP respectively. The recombinant sites were identified by the flanking markers. Orange frame and blue arrows indicate the location of *qWS8/ipa1-2D* repeats and *IPA1* respectively. (**b**) Semi-quantitative RT-PCR detection of *IPA1* expression in IM tissues of recombinant sibling lines. Rice *Ubiquitin* cDNA was amplified as internal control. The cycle number of PCR amplification is 23 both for *IPA1* and *Ubiquitin*.

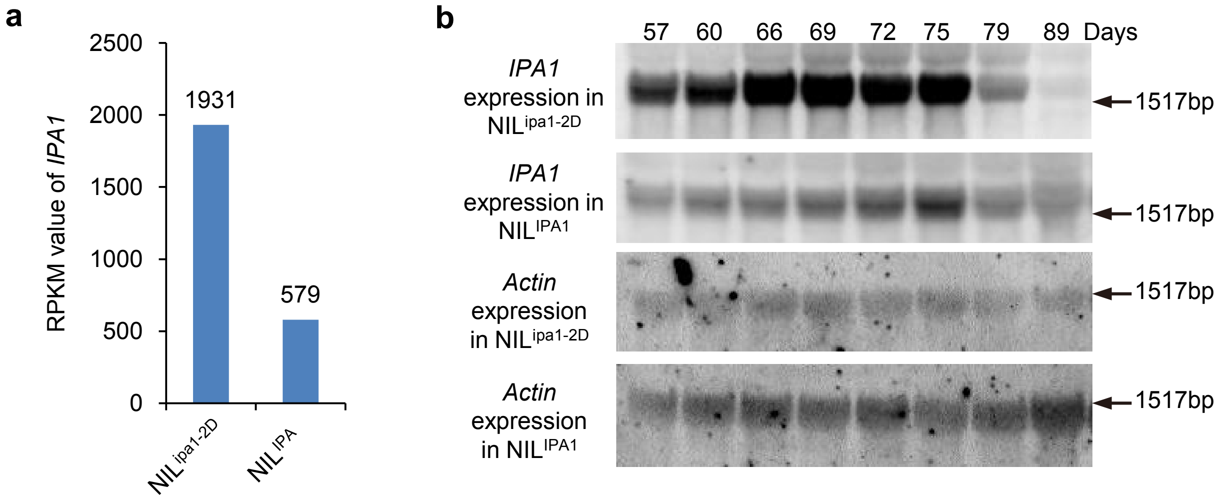

Supplementary Figure 9 Comparison of *IPA1* expression between NIL$^{ipa1\text{-}2D}$ and NIL^{IPA1}. (**a**) Total RNAs prepared from IM tissues were subjected to RNA-sequencing, and RPKM (Reads per kilobase per million reads) values of *IPA1* are shown. (**b**) Northern blotting shows transcript levels of *IPA1* in IMs of sequential developmental stages in NIL$^{ipa1\text{-}2D}$ and NIL^{IPA1}. The numbers in the upper panel denote the IM stages corresponding to days after germination. Rice *Actin* mRNA was probed as internal control.

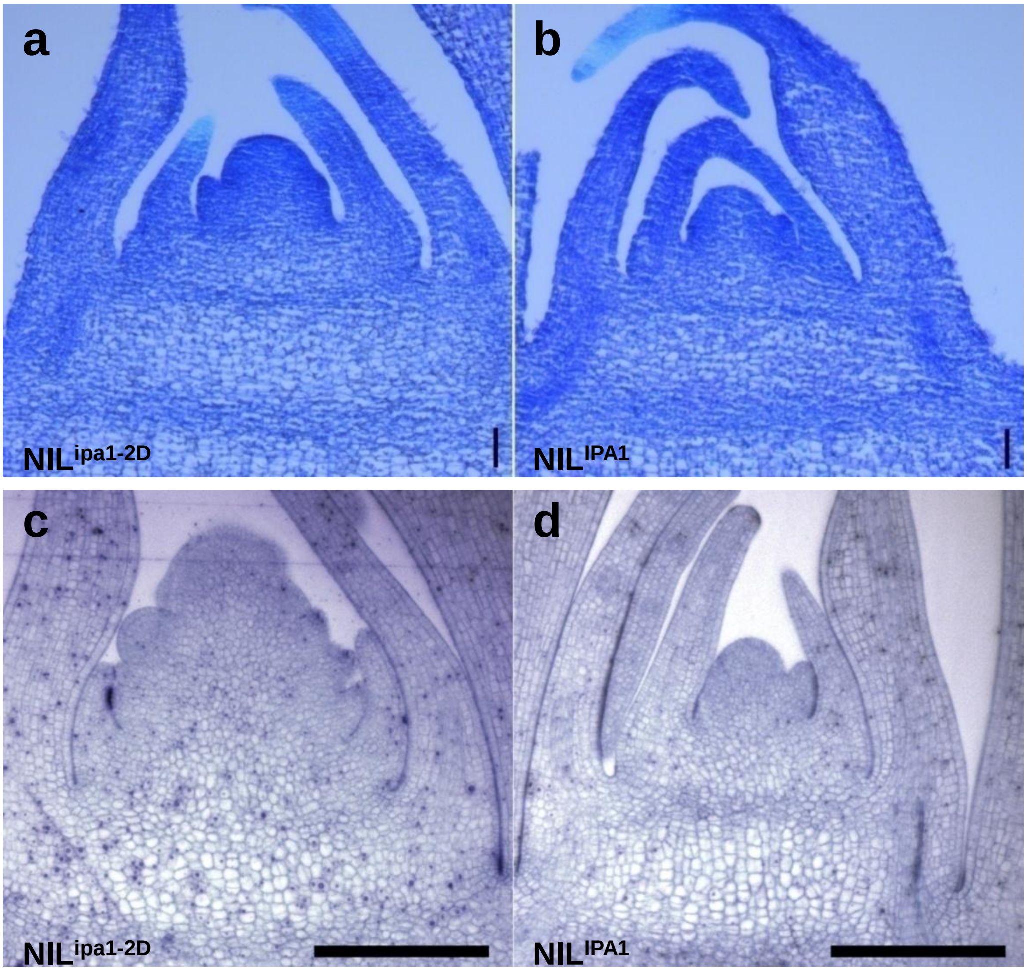

Supplementary Figure 10 Inflorescence meristems development in NILs. (**a,b**) Section of IMs of NIL$^{ipa1\text{-}2D}$ (**a**) and NIL^{IPA1} (**b**) in paraffin at IM1 stage. Scale bars, 50 µm. (**c,d**) Section of IMs of NIL$^{ipa1\text{-}2D}$ (**c**) and NIL^{IPA1} (**d**) in resin at IM2 stage. Scale bars, 200 µm. Note that the IM of NIL$^{ipa1\text{-}2D}$ develops bigger than that of NIL^{IPA1}.

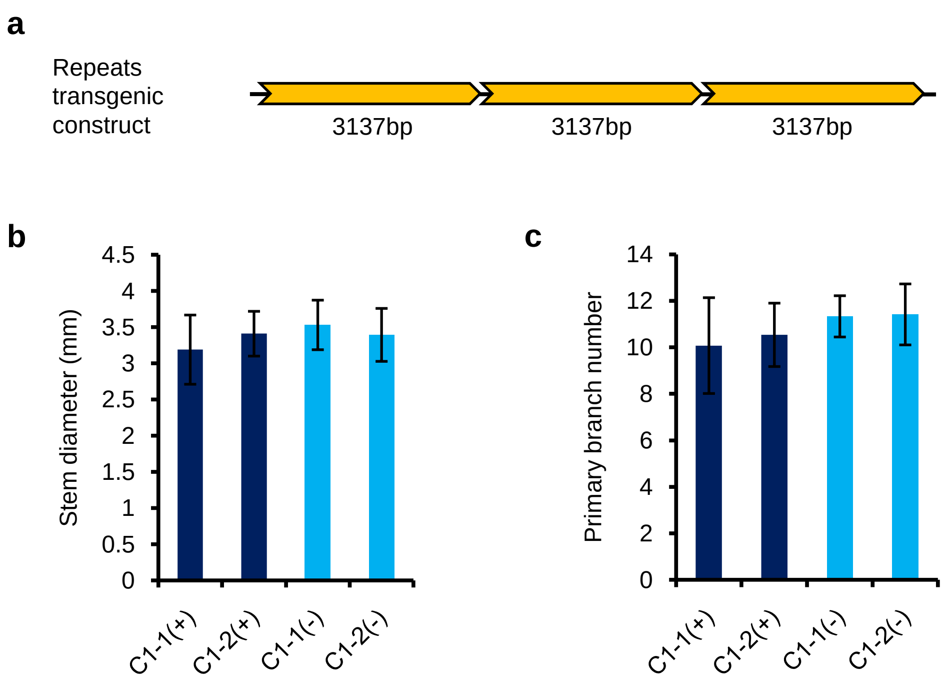

Supplementary Figure 11 Phenotypes of transgenic plants with the three tandem repeats of *ipa1-2D*. (**a**) The tandem repeats were cloned into vector pCambia1300 and transformed into wild-type NIP callus. (**b,c**) Comparison of stem diameter (**b**) and panicle primary branch number (**c**) in transgenic positive (+) and negative (-) lines. Values are means ± s.d. (*n* = 9). No significant difference was detected by Tukey HSD test, indicating the tandem repeats do not have *trans*-acting function.

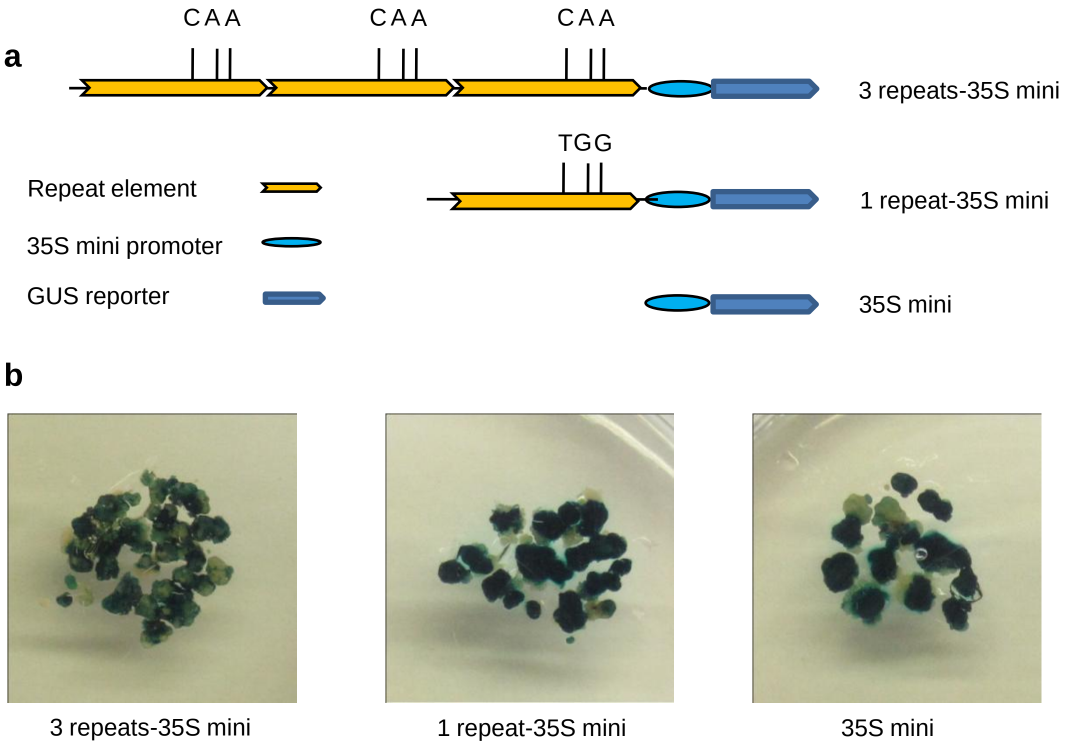

Supplementary Figure 12 The repeat element does not display enhancer activity. (**a**) Schematic map of constructs with tandem repeats or single repeat fused with the 35Smini promoter and GUS. The constructs were transformed into NIP callus respectively. (**b**) GUS staining of transgenic callus expressing the fusion reporters did not show difference of reporter expression.

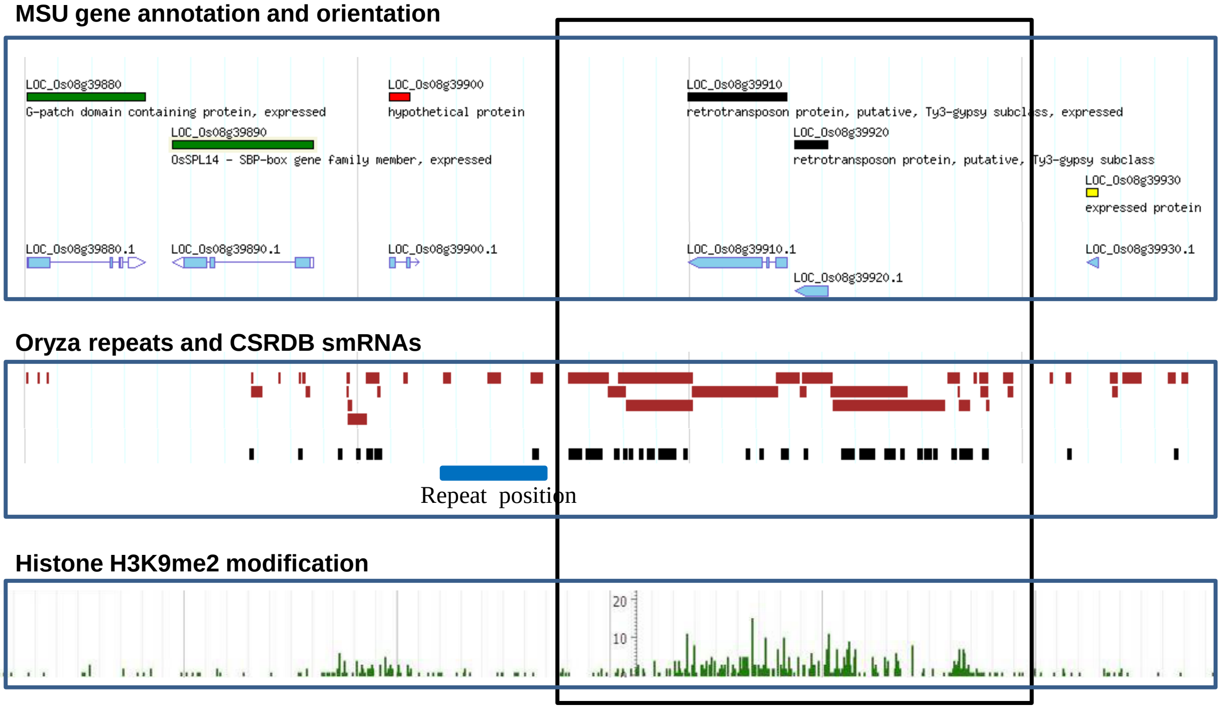

Supplementary Figure 13 Genomic structure and gene annotation of the chromosomal region flanking *IPA1* in the NIP reference genome. The upper panel shows the gene annotation and genomic structure by Michigan State University (MSU); middle panel displays repeat (red boxes) and small RNA (black boxes) regions predicted by Cereal Small Database (CSRDB), and the position of the *qWS8/ipa1-2D* tandem repeats is labelled by blue boxes; lower panel displays regions of H3K9me2. The black frame indicates the region enriched for TE elements and H3K9me2 modification.

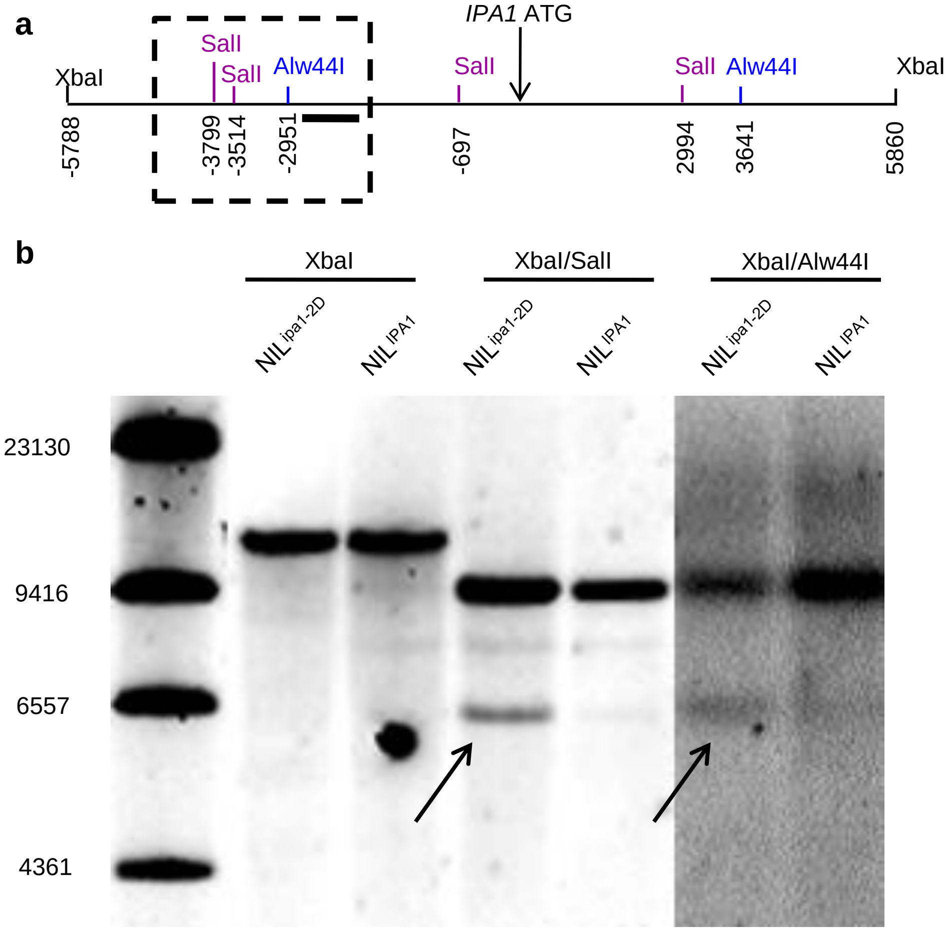

Supplementary Figure 14 Methylation analysis of the region between the repeats and *IPA1*. (**a**) Schematic map indicating enzymes used with positions from *IPA1* ATG (0). Black bar denotes position of probe used for Southern blot and dotted frame denotes the position with methylation sensitivity of enzyme digestion according to the bind size in **b**. (**b**) Southern blot of $NIL^{IPA1\text{-}2D}$ and NIL^{IPA1}. The gDNA digested with XbaI was used as a control. The difference in methylation pattern is denoted by arrows.

TCTTCCGGGCAGAGCTCTCGAAGCTCGCCCTCCTCGCGCGCCGGTGGCGTTGGCGGGGCCCGCGTGTGGC

TACGCAGCTCCGGTGCTGCGCCTCCACCGTCGACGACAGCGCCGCTTGGCGCTGCCGCCGTCTTCCGCCGC

GCCGCTGGACGCCGCCAGATCTGCTGCTCGTCGCCGCGTGGGCCGCTCCACCCGGTTGGAGGAGGAGAGG

CGGCGCCGCGCTTGGGCTGCCCCACCGCCGAGCTCTGCCGCGCCGTTCGCCGGTGCTGCCGAGCTCCGCC

GCGCCTGCCGGAGCACGCTGCCATGGCCGCCCTGGAGAAGACACGAGAGAATTAGGTGGAGGGTGGGGG

AAGGGTGAGATTTTTTATATTATCTATGGGTC/TCCATTTATAAATTTTCTAAACCACACTTATACTGTGGGTGC

AGTGTCATTTAGAGTTCCCAAACCACCTATGTTGCAGCTGTGGTATAACAATTTGCTAGGACGCATTGCTACT

GCCCTTGTACCCTGCTATAAGAAGATAACCAATGACATCTCCACTCGATTTTCTCGGCGCGCGTGTGAGGGT

GTGAGGATAATTTTTATTTTAAGTGGTTTTTAAGGGCGGAGAGAGAGAGAGAGAGGGCACCGCACTAC

TTCTACTTGTGTGTGTGTCGCTCGCTGGGCTTCGCCACCTTTCCGTCTCTTTCCTCTCTCTTCTCTCTCCCCCTC

TCCTGGAGGAGAGAGAGGAGAAGAGGAGGGGGGCCGCGCCAAGAGCCACGCGCGCTACAGTCTCCTT

CCCACCCGCGACCGCGAGCA*ATG*

Supplementary Figure 15 Sequence of the 800-bp promoter region from *IPA1* ATG. The sequence of DH (DNase I hypersensitive) site is indicated by blue letters, and two TATA boxes and CAAT boxes are highlighted by the black frames. Letters with yellow shade denote the SNP 419 bp from ATG (0). The underlined letters indicated the junction region with obvious methylation difference between *ipa1-2D* and *IPA1* allele.

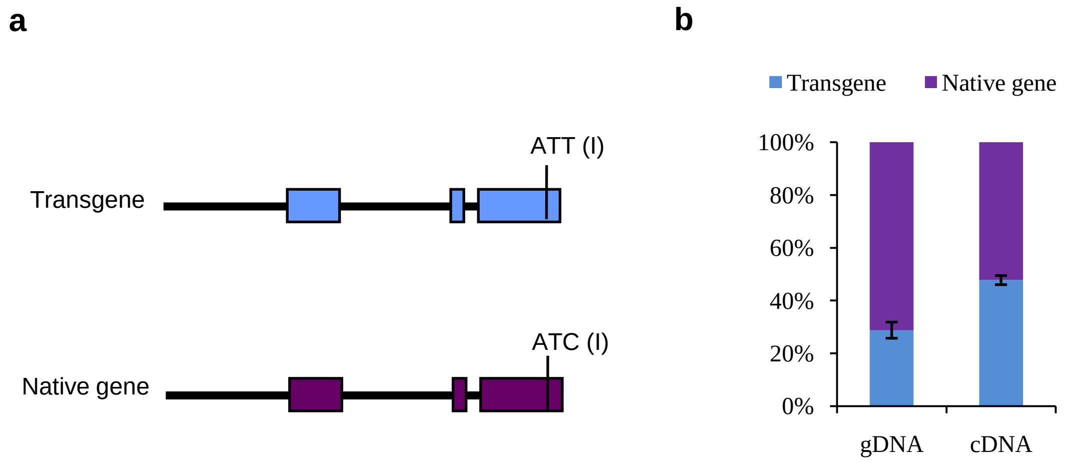

Supplementary Figure 16 Comparison of gDNA and mRNA from transgene and native gene. (**a**) An 8-kb *IPA1* genomic DNA with an artificial SNP (from ATC to ATT) that does not alter the amino acid (in parenthesis) was transformed into NIP to generate transgenic plants. (**b**) Ratio of gDNA and mRNA in the T_0 plants with the transgenic construct. Alleles from transgenic construct and native *IPA1* were discriminated by sequencing the colonies of PCR or RT-PCR products covering the SNP and indicated by blue and purple colors respectively. Values are means ± s.d. obtained from four independent transgenic plants.

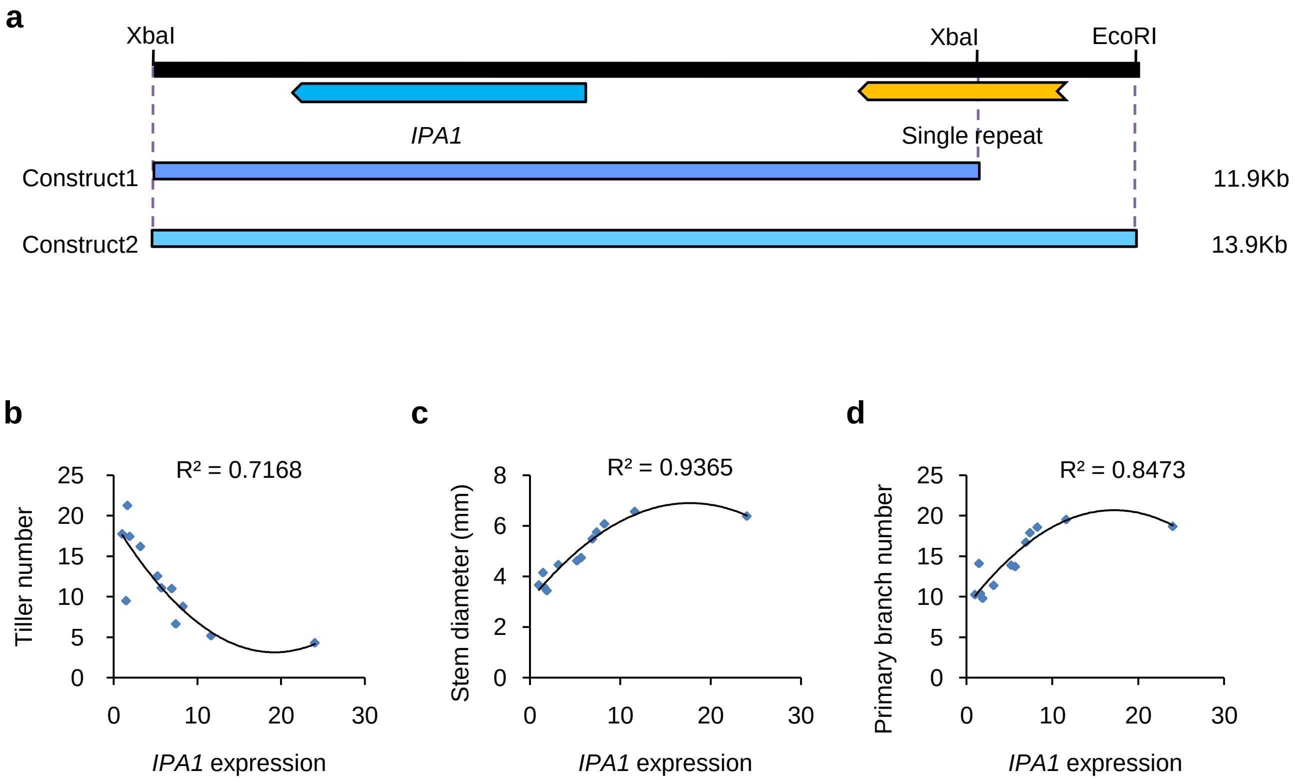

Supplementary Figure 17 Dose effect of *IPA1* on IPA traits shown by transgenic plants with different *IPA1* gDNA constructs. (**a**) Schematic map of two *IPA1* gDNA transgenic constructs. Construct 2 with 13.9-kb genomic region contains the whole *IPA1* and the upstream region with a single repeat, and construct 1 with 11.9 kb contains *IPA1* and only half of the repeat. Both constructs were transformed into NIP to generate stable transgenic plants. (**b-d**) Plots of relative *IPA1* expression levels with (**b**) tiller number, (**c**) stem diameter and (**d**) panicle primary branch number. Data were obtained from twelve independent lines with construct 2. Curves fitting the traits change are calculated by quadratic equation with an R^2 values.

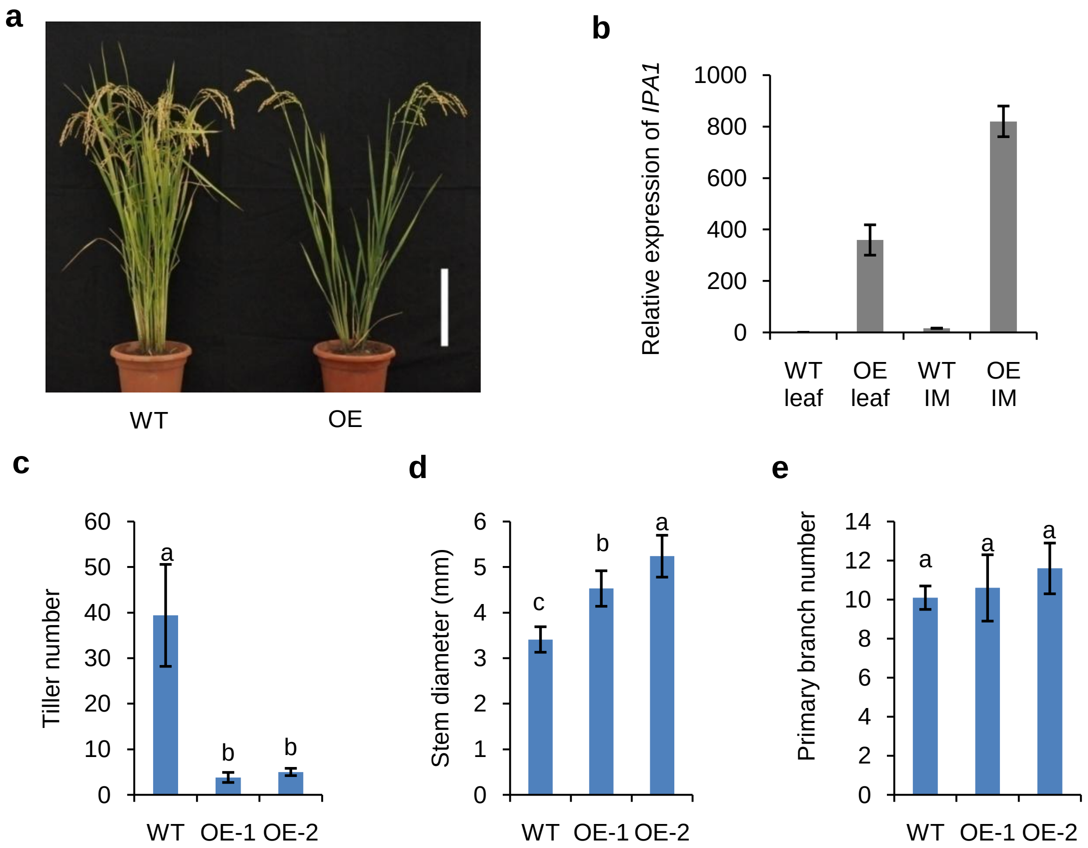

Supplementary Figure 18 Analysis of transgenic plants ectopically over-expressing *IPA1* by the CaMV35S promoter. (**a**) Morphology of wild type (WT) plant and over-expression (OE) plant. Scale bar, 20cm. (**b**) Relative expression levels of *IPA1* in leaf and IM of WT and OE plants, normalized to rice *Actin*. Values are means ± s.d. (n = 3). (**c-e**) Comparison of tiller number (**c**), stem diameter (**d**) and panicle primary branch number (**e**) among WT and OE lines. Values are means ± s.d. (n = 8). Different letters at top of each column indicate a significant difference at $P < 0.05$ determined by Tukey HSD test. Note that ectopic over-expression of *IPA1* did not stimulate panicle development.

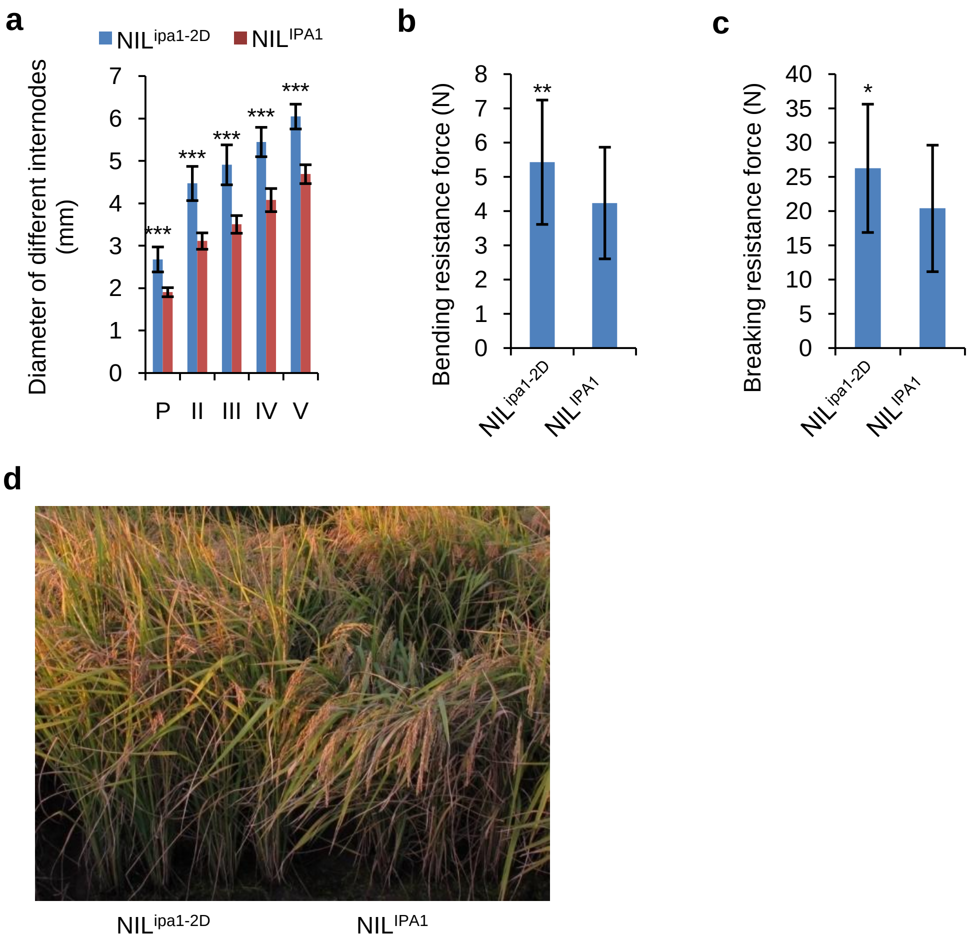

Supplementary Figure 19 Comparison of stem diameter and lodging resistance between NILs. (**a**) Stem diameter of five internodes of NIL$^{ipa1\text{-}2D}$ and NIL^{IPA1} (P, peduncle, and other 4 internodes). (**b**) Bending resistance and **(c**) breaking resistance of NIL$^{ipa1\text{-}2D}$ and NIL^{IPA1}. Values in (**a**-**c**) are means $\pm$ s.d. (n = 12 for **a**, 46 for **b** and 30 for **c**). Significant difference between NILs is determined by the Student's *t*-test (* $P < 0.05$, ** $P < 0.01$ and *** $P < 0.001$). (**d**) Plants of NIL$^{ipa1\text{-}2D}$ and NIL^{IPA1} grown in the Shanghai station after the strong typhoon in the 2013 summer, indicating that NIL$^{ipa1\text{-}2D}$ showed better performance of lodging resistance than NIL^{IPA1}.

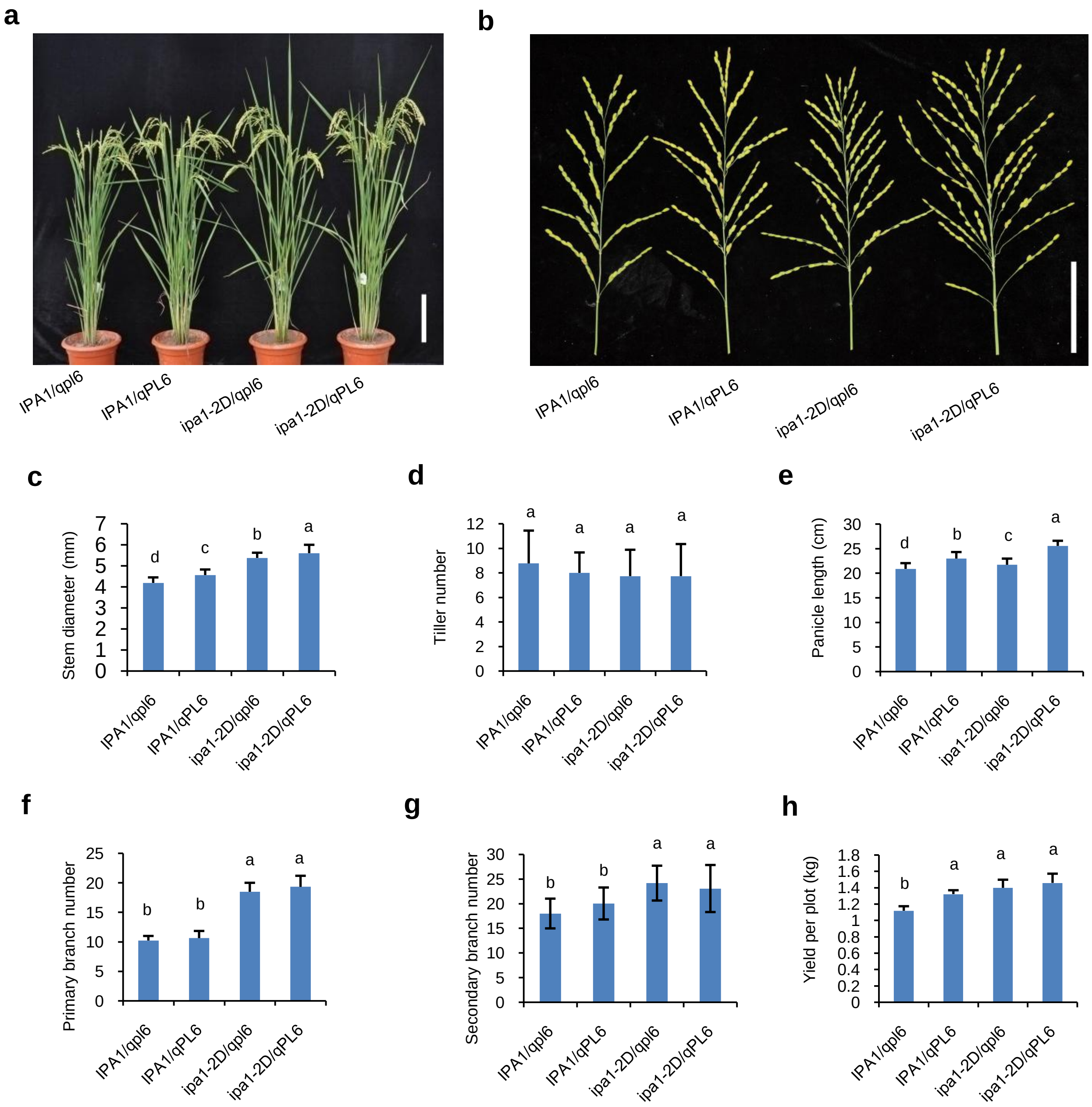

Supplementary Figure 20 Genetic effect of pyramiding *ipa1-2D* with *qPL6*. (**a**) Morphology of plants with different allele combinations of *ipa1-2D* with *qPL6* that controls panicle length. Scale bar, 20 cm. (**b**) Panicle morphology of plants with combinations of two loci as shown in (**a**). Scale bar, 9 cm. (**c-h**) Yield-related traits of plants, including stem diameter (**c**), tiller number (**d**), panicle length (**e**), panicle primary branch number (**f**), panicle secondary branch number (**g**) and grain yield per plot (2.5 m^2) (**h**). Values in **c-h** are means ± s.d (n = 30 for **c-g** and 6 for **h**). Different letters at top of each column indicate a significant difference at $P < 0.05$ determined by Tukey HSD test. Note that yield performance of plants pyramiding *ipa1-2D* with *qPL6* was further improved though statistically not significant.

Supplementary Table 1 QTL detected from F_2 population of YYP1/NIP

Traits	Chr	Interval	LOD	Additive effect	Dominant effect	R^2
Major axis diameter of internode 3	3	RM6849-RM1022	2.7	-0.04	0.44	4%
Major axis diameter of internode 3	3	RM5626-RM1350	3.1	0.21	-0.19	5%
Major axis diameter of internode 3	5	RM5642-RM1054	4.2	0.31	0.18	3%
Major axis diameter of internode 3	6	RM1340-RM5814	3.6	0.32	0.12	4%
Major axis diameter of internode 3	8	RM284-RM5814	2.7	-0.16	0.39	6%
Major axis diameter of internode 3	8	RM3452-RM3120	23.4	0.84	0.33	21%
Minor axis diameter of internode 3	2	RM7451-RM5897	3.2	-0.05	0.34	4%
Minor axis diameter of internode 3	5	RM5642-RM1054	3.9	0.29	0.04	6%
Minor axis diameter of internode 3	8	RM3452-RM3120	18.4	0.69	0.14	24%
Major axis diameter of internode 4	1	RM1282-RM1167	3.2	0.27	0.01	5%
Major axis diameter of internode 4	5	RM5642-RM1054	5.5	0.33	0.17	4%
Major axis diameter of internode 4	8	RM1235-RM6429	3.7	0.42	0.33	5%
Major axis diameter of internode 4	8	RM284-RM5814	4.9	-0.28	0.38	13%
Major axis diameter of internode 4	8	RM3452-RM3120	18.7	0.81	0.21	19%
Minor axis diameter of internode 4	2	RM7451-RM5897	3.3	0.07	0.41	1%
Minor axis diameter of internode 4	5	RM5642-RM1054	3.8	0.23	0.20	2%
Minor axis diameter of internode 4	7	RM11-RM18	2.6	0.12	-0.28	5%
Minor axis diameter of internode 4	8	RM1235-RM6429	2.8	0.36	0.23	6%
Minor axis diameter of internode 4	8	RM284-RM5814	3.8	-0.23	0.35	12%
Minor axis diameter of internode 4	8	RM3452-RM3120	15.6	0.68	0.22	15%

Supplementary Table 2 Genotype and phenotype of 188 IPA stocks

Stocks	*qWS8* repeat	SNP Haps[a]	Hainan PBN[b] (n=3)	Zhejiang PBN (n=5)	Hainan SD[c] (mm,n=3)	Zhejiang SD (mm,n=5)
I1	-	Hap4	10.3	17.8	4.75	6.28
I2	-	Hap4	10.7	19.6	5.35	6.35
I3	-	Hap4	10.7	15.8	4.99	5.57
I4	-	Hap4	8.5	16.8	5.00	5.58
I5	-	Hap1	11.7	14.2	5.30	5.26
I6	-	Hap4	N.D.	14.0	5.01	5.27
I7	-	Hap4	7.0	13.2	4.77	5.00
I8	-	Hap4	7.0	12.8	4.62	5.26
I9	-	Hap4	10.0	15.0	5.29	5.25
I10	-	Hap4	9.7	17.8	6.23	5.95
I11	-	Hap4	9.3	14.3	4.68	4.79
I12	-	Hap4	10.0	15.4	4.65	4.98
I13	-	Hap1	16.3	20.4	6.31	5.56
I14	-	Hap4	10.0	17.8	5.62	5.77
I15	-	Hap4	9.3	18.2	5.99	5.86
I16	-	Hap4	N.D.	18.2	5.58	6.23
I17	-	Hap4	9.0	18.2	N.D.	5.52
I18	-	Hap4	8.7	16.8	5.94	5.24
I19	-	Hap4	9.7	19.0	6.04	6.26
I20	-	Hap4	10.7	19.0	6.26	6.71
I21	-	Hap4	13.0	19.8	5.82	6.61
I22	-	Hap4	11.0	16.8	6.70	6.63
I23	-	Hap4	10.7	18.0	6.94	6.88
I24	-	Hap4	10.7	16.6	6.10	6.31
I25	+	Hap10	15.3	28.5	7.93	7.71
I26	-	Hap4	10.7	18.0	5.28	4.82
I27	-	Hap4	8.7	21.6	5.17	5.04
I28	-	Hap4	6.3	11.8	4.17	4.44
I29	-	Hap4	12.3	17.8	6.92	6.73
I30	-	Hap4	11.3	17.2	7.01	6.61
I31	+	Hap10	14.0	26.4	7.48	7.75
I32	-	Hap4	11.0	17.8	7.00	7.00
I33	-	Hap4	11.0	15.8	7.16	7.30
I34	-	Hap4	14.7	18.6	6.29	6.14
I35	-	Hap4	9.3	13.6	5.36	4.57
I36	-	Hap4	N.D.	16.8	N.D.	5.45
I37	-	Hap4	12.0	17.6	7.12	7.85
I38	+	Hap10	14.3	22.4	7.45	7.22
I39	-	Hap4	N.D.	19.6	N.D.	4.67
I40	+	Hap10	13.0	25.2	5.91	7.19
I41	+	Hap10	14.7	27.2	6.26	7.21

I42	-	Hap4	10.3	13.4	4.91	5.91
I43	-	Hap4	9.0	17.4	6.20	6.63
I44	+	Hap10	24.3	28.4	9.33	9.41
I45	-	Hap4	11.0	17.6	6.01	5.41
I46	-	Hap4	N.D.	18.6	N.D.	5.40
I47	-	Hap4	10.0	18.8	5.46	5.48
I48	-	Hap4	11.0	17.2	6.57	7.53
I49	-	Hap4	9.3	17.4	6.63	7.90
I50	-	Hap4	11.3	16.0	6.65	7.79
I51	-	Hap4	11.0	16.2	6.89	7.39
I52	-	Hap4	11.7	17.2	7.31	7.40
I53	-	Hap4	11.3	17.4	7.11	7.35
I54	-	Hap4	12.0	17.4	6.46	7.27
I55	-	Hap4	14.3	17.4	5.49	5.84
I56	-	Hap4	N.D.	17.8	5.10	5.76
I57	-	Hap4	N.D.	13.4	N.D.	4.83
I58	-	Hap4	N.D.	14.8	N.D.	4.62
I59	-	Hap4	8.7	15.4	4.64	4.13
I60	-	Hap4	N.D.	18.6	N.D.	6.97
I61	-	Hap1	9.0	14.8	4.83	6.01
I62	-	Hap1	9.3	14.0	4.97	6.16
I63	-	Hap1	10.3	14.6	4.93	6.42
I64	-	Hap1	11.0	16.4	5.01	5.56
I65	+	Hap10	13.0	21.6	6.93	8.12
I66	+	Hap10	13.0	16.0	7.57	7.04
I67	+	Hap10	12.0	17.6	6.42	6.77
I68	+	Hap10	13.0	18.4	6.63	7.05
I69	+	Hap10	13.0	17.4	6.99	6.14
I70	+	Hap10	14.3	17.8	6.00	6.90
I71	+	Hap10	20.7	32.4	7.66	9.87
I72	+	Hap10	24.3	37.4	7.20	8.54
I73	+	Hap10	20.3	31.4	7.83	9.04
I74	+	Hap10	18.0	N.D.	7.47	8.86
I75	+	Hap10	12.7	N.D.	6.31	N.D.
I76	+	Hap10	17.7	32.6	7.49	8.16
I77	-	Hap1	9.7	11.0	5.14	5.62
I78	-	Hap1	14.7	18.0	6.29	7.72
I79	-	Hap1	9.7	15.0	N.D.	5.71
I80	+	Hap10	22.3	28.8	7.98	8.93
I81	-	Hap1	8.7	13.6	4.78	6.26
I82	+	Hap10	24.3	28.4	9.23	N.D.
I83	-	Hap1	16.0	17.2	6.63	7.50
I84	-	Hap1	10.0	13.6	5.43	5.83
I85	-	Hap1	13.3	17.6	6.37	7.31

I86	-	Hap4	12.3	14.6	5.76	5.70
I87	-	Hap4	15.0	21.0	6.57	7.18
I88	-	Hap1	10.7	16.0	5.68	6.31
I89	+	Hap10	15.7	N.D.	6.03	8.56
I90	+	Hap10	15.3	N.D.	6.91	N.D.
I91	+	Hap10	21.0	24.8	7.98	7.73
I92	+	Hap10	18.0	21.6	6.58	8.16
I93	+	Hap10	21.0	35.6	7.50	8.81
I94	+	Hap10	16.7	20.4	6.91	8.45
I95	-	Hap4	9.0	14.0	4.08	4.23
I96	+	Hap10	N.D.	21.2	N.D.	8.10
I97	+	Hap10	18.0	24.8	7.69	7.93
I98	+	Hap10	N.D.	24.4	7.37	8.07
I99	+	Hap10	17.7	22.6	7.73	8.48
I100	+	Hap10	27.0	34.8	7.66	8.12
I101	+	Hap10	17.0	22.2	6.99	8.28
I102	+	Hap10	18.0	22.0	7.13	7.59
I103	+	Hap10	17.7	21.6	7.06	8.78
I104	+	Hap10	22.0	27.2	7.17	7.79
I105	-	Hap4	9.0	12.6	4.12	4.04
I106	+	Hap10	17.0	20.8	6.82	7.98
I107	-	Hap4	8.0	12.2	4.14	4.45
I108	+	Hap10	19.0	27.4	6.77	7.51
I109	+	Hap10	21.0	28.8	8.16	8.97
I110	+	Hap10	18.3	25.5	7.48	8.91
I111	+	Hap10	17.3	22.4	8.11	8.09
I112	+	Hap10	18.7	22.8	6.94	6.77
I113	+	Hap10	27.0	40.4	8.28	8.21
I114	+	Hap10	17.0	22.6	7.31	7.07
I115	+	Hap10	20.0	21.2	7.93	8.10
I116	+	Hap10	13.0	21.4	7.74	8.29
I117	+	Hap10	13.3	22.8	6.69	7.94
I118	+	Hap10	15.0	21.4	6.92	8.12
I119	-	Hap4	7.7	13.2	3.75	4.14
I120	+	Hap10	16.0	22.0	7.72	8.19
I121	+	Hap10	16.0	22.6	7.32	8.04
I122	+	Hap10	17.0	20.4	6.95	8.34
I123	+	Hap10	18.0	22.8	7.90	8.56
I124	+	Hap10	17.3	22.0	7.09	8.24
I125	+	Hap10	20.0	28.0	7.96	8.79
I126	+	Hap10	13.0	21.4	7.27	8.02
I127	+	Hap10	12.7	20.2	7.37	7.89
I128	+	Hap10	14.0	25.2	7.20	7.56
I129	+	Hap10	17.3	21.2	7.01	7.98

I130	+	Hap10	17.3	25.4	7.62	8.24
I131	+	Hap10	17.3	23.6	7.15	9.95
I132	-	Hap1	9.0	13.4	4.23	4.53
I133	+	Hap10	17.7	22.4	7.80	8.89
I134	+	Hap10	11.3	22.6	6.77	7.76
I135	+	Hap10	15.0	20.4	5.38	6.66
I136	+	Hap10	25.3	32.0	7.97	9.08
I137	+	Hap10	16.0	24.4	7.16	8.78
I138	+	Hap10	26.3	37.8	N.D.	9.44
I139	+	Hap10	15.3	22.2	6.98	8.42
I140	-	Hap1	11.0	13.6	5.37	4.83
I141	-	Hap4	9.0	12.0	3.89	4.57
I142	+	Hap10	16.7	18.8	6.60	7.07
I143	-	Hap1	14.7	18.0	6.09	8.62
I144	-	Hap1	12.0	18.0	4.85	7.08
I145	-	Hap4	8.3	14.8	5.07	4.99
I146	-	Hap4	11.7	14.8	5.92	6.15
I147	+	Hap10	N.D.	26.2	N.D.	7.23
I148	-	Hap1	9.3	11.4	4.59	5.00
I149	+	Hap10	21.3	38.2	7.39	7.98
I150	-	Hap4	7.3	11.6	5.33	5.54
I151	-	Hap4	11.0	14.2	5.93	6.70
I152	-	Hap1	10.7	14.4	6.52	6.64
I153	-	Hap1	17.0	22.4	6.02	7.18
I154	+	Hap10	19.0	24.8	7.99	8.57
I155	+	Hap10	12.0	19.0	6.79	7.71
I156	-	Hap1	11.7	13.4	4.77	4.73
I157	+	Hap10	22.7	34.8	7.67	9.51
I158	+	Hap10	16.3	22.4	7.59	8.83
I159	+	Hap10	16.3	22.8	7.73	8.94
I160	+	Hap10	25.0	30.2	8.12	9.36
I161	+	Hap10	21.3	34.2	8.21	9.58
I162	+	Hap10	15.7	23.8	N.D.	9.52
I163	+	Hap10	15.7	21.2	7.44	9.04
I164	+	Hap10	23.7	29.6	7.82	8.75
I165	+	Hap10	21.7	32.8	7.67	9.36
I166	+	Hap10	19.0	23.5	7.46	8.62
I167	+	Hap10	16.7	24.6	6.85	8.26
I168	+	Hap10	18.0	20.2	6.87	8.01
I169	+	Hap10	N.D.	30.4	7.99	9.46
I170	+	Hap10	19.0	23.0	7.12	8.51
I171	+	Hap10	17.0	21.4	7.48	7.83
I172	+	Hap10	16.0	22.6	7.25	9.18
I173	+	Hap10	22.7	30.0	7.74	9.46

I174	+	Hap10	26.0	31.5	7.71	9.47
I175	+	Hap10	18.0	21.2	6.63	7.24
I176	+	Hap10	21.0	N.D.	6.65	N.D.
I177	+	Hap10	18.0	22.2	6.54	7.49
I178	+	Hap10	20.7	21.2	6.78	7.06
I179	+	Hap10	23.7	20.4	N.D.	7.00
I180	+	Hap10	20.0	24.2	6.64	7.34
I181	+	Hap10	17.0	25.0	7.04	7.78
I182	+	Hap10	20.3	25.4	7.53	7.54
I183	+	Hap10	19.0	29.0	8.24	8.88
I184	-	Hap1	11.7	14.0	5.77	5.95
I185	-	Hap1	11.0	N.D.	5.27	N.D.
I186	+	Hap10	22.3	N.D.	6.58	N.D.
I187	+	Hap10	17.0	34.8	6.71	7.56
I188	+	Hap10	23.0	38.2	6.66	8.46

[a]corresponds to the SNP haplotypes in supplementary figure 6

[b]Panicle primary branch number

[c]Stem diameter

N.D., not determined

Supplementary Table 3 Mid-parent heterosis of six yield related traits

	Tiller number	1000-grain weight	Panicle primary branch number	Panicle secondary branch number	Spikelet number per panicle	Yield per plant
Mid-parents heterosis rate in Shanghai	-0.02	-0.01	0.02	0.11	0.07	0.06
Mid-parents heterosis rate in Hainan	-0.08	0.01	-0.01	0.16	0.04	0.03

Supplementary Table 4 Yield test of newly-developed varieties with *ipa1-2D* and *ipa1-1D* alleles

Varieties	Alleles type	Zhejiang			Hainan		
		Tiller number ($667m^2$)	Yield ($kg/667m^2$)	Increased percentage	Tiller number ($667m^2$)	Yield ($kg/667m^2$)	Increased percentage
JYZK-3	*IPA1-2D*/*IPA1*	1.641×10^5	971.62	31.10%	1.463×10^5	642.9	23.92%
JYZK-4	*IPA1-2D*/*IPA1*	1.641×10^5	960.88	29.65%	1.482×10^5	631.3	21.68%
JYZK-6	*IPA1-1D*/*IPA1*	1.294×10^5	918.31	23.91%	1.248×10^5	616.6	18.85%
JYZK-33	*IPA1-1D*/*IPA1*	1.275×10^5	925.46	24.87%	1.247×10^5	622.6	20.01%
JY-5	*IPA1*/*IPA1*	1.575×10^5	741.11		1.69×10^5	518.8	

Supplementary Table 5 Primers used in this study

Names	Primer sequence (5'-3')	Enzymes used
Primers of PCR-based molecular markers		
SnpM1-F	TGAACGTGTTCCATAGCTTCAC	HaeIII
SnpM1-R	AGGCATGCAAGTCCATTCTTAT	
SnpM2-F	AGGGTGAGATTTTTTATATTATCTATGGAT	BamHI
SnpM2-R	TCTCTCTCTCTCTCTCTCCG	
SnpM3-F	GTTCAAAGTACATGTAGTTTTGTGATTAA	VspI
SnpM3-R	TAGGTCTTGGAGTCTGTGCTGC	
SnpM4-F	TACCACGTGGGAACCGTGCTTACCGCCCG	BcnI or HpaII
SnpM4-R	GCTCCAAAGATGACTGGCCTTGCCGCT	
SnpM5-F	GGGCGTAGTAACCCTGATCCCGACGGTTC	AsuII
SnpM5-R	TGGAGGCAGGTCCTGTTGCCCTTGCTGT	
SnpM6-F	CCAGTTATCAAATCACGCAGAA	Tsp45I
SnpM6-R	GTTACAACCCTCCACCATTCAC	
SnpM7-F	ACCAACGTCCTGGATAGCGTACTGTCAGG	HaeIII
SnpM7-R	GCTTAGCCCCAGTTATCTTTTGTTTTTC	
InDelM-F	TATAGATGTTTGAGATTCGTCC	
InDelM-R	AAATGAACTGGCTAGATTGG	
Repspecific-F	ACAGAGCCTCCATATCTCAG	
Repspecific-R	GGTAGCAGCACACTATTCCT	
Probes for Southern and Northern blot		
IPA1UpProbe1-F	AGGGTTGTACCACTGGTAAA	
IPA1UpProbe1-R	CCATCGTCGGAAAGGGATTT	
IPA1UpProbe2-F	GGTTTAAGTTTGTGTTCCCC	
IPA1UpProbe2-R	CCGAATTTATATGAGCTGCTA	
IPA1-Probe-F	CCAAGCAGCGTAAGGAATG	
IPA1-Probe-R	TTGAACACAAAATAAGGGCA	
Actin-Probe-F	TACTCATTCACCACAACGG	
Actin-Probe-R	TCGCAACTCAAAAACAACC	
Hpt-Probe-F	CTCCATACAAGCCAACCACG	
Hpt-Probe-R	AAAAGCCTGAACTCACCGC	
Primers used for RT-PCR and real-time PCR		
IPA1-RT-F	ATTTGACCAAGGAAAACGCA	
IPA1-RT-R	AGACATCCACCTGGAGGGAG	
Ubi-RT-F	GACGGACGCACCCTGGCTGACTAC	
Ubi-RT-R	TGCTGCCAATTACCATATACCACGAC	
IPA1-qRT-F	GACTAGCTGCATCTGTTGGTGAGC	
IPA1-qRT-R	TGCTGGCCATGCATTCCTTACG	
Actin-qRT-F	CGGGAAATTGTGAGGGACAT	
Actin-qRT-R	AGGAAGGCTGGAAGAGGACC	
miR156-qRT-F	TGACAGAAGAGAGTGAGCAC	
miR529p-qRT-F	AGAAGAGAGAGAGTACAGCC	
5SrRNA-qRT-F	AAACACCCGATCCCATTCC	

5SrRNA-qRT-R	GGCTTTGACCATGTCTCCC
Primers used to generate DNA constructs	
ThreeReps-F	ATCCATCAGCACAACAGAG
ThreeReps-R	AAAAACAAATACATAGCAAAGT
OneReps-F	AGTGGGTCCCGTGTGTCAGT
OneReps-R	CTCGTCGTCAACCATTGCC
35SminiPro-F	ACTAGTATCTCCACTGACGTAAGGGA
35SminiPro-R	GGATCCTTCTCTCCAAATGAAATGAA
IPA1SNPmut-F	AGCCCCAGCACCACGCATtGATCCTGGGTC
IPA1SNPmut-R	AATGCGTGGTGCTGGGGCTGGACCGTTCCC
Primers used for BSP sequencing, transgene ratio detection and chromatin openness detection	
BSPSeq1-F1	GGTTCGTCGGAGTAGGGG
BSPSeq1-R1	ATATCATTAATTATCTTCTTAT
BSPSeq1-F2	TAGGGGCGTTCGGGGAGTTTT
BSPSeq1-R2	TTTAACAAAATACAAAACAATAA
BSPSeq2-F	TGTGGGTGYAGTGTYATTTAGAGTT
BSPSeq2-R	CCTCCTCCACTRRCCATCTCCATT
IPA1cDNA -F	ATCTGAGCGGGATCAAGAACT
IPA1cDNA -R	CATCGTGTTGCTGGTTTGG
IPA1gDNA -F	AACACAAAATAAGGGCAGGC
IPA1gDNA -R	AGGGTTCCAAGCAGCGTAAG
ChromatinMn1-F	TTGCTGGTTTGGTCGAAGGT
ChromatinMn1-R	TGGATGTCTCGCAGGGGTC
ChromatinMn2-F	GCCTCTTATCCATCAGCACAA
ChromatinMn2-R	CCATCGGTAGCAGCACACTAT
ChromatinMn3-F	GCGGGTGGGAAGGAGACTGT
ChromatinMn3-R	CGCCCTGGAGAAGACACGAGA
ChromatinMn4-F	CGGGGTACACACACGACAAAG
ChromatinMn4-R	ATGGGCGATCAACCAGATTC
